# A novel cell-printing method and its application to hepatogenic differentiation of human adipose stem cell-embedded mesh structures

**DOI:** 10.1038/srep13427

**Published:** 2015-08-21

**Authors:** Seung Hyun Ahn, Hyeong Jin Lee, Ji-Seon Lee, Hyeon Yoon, Wook Chun, Geun Hyung Kim

**Affiliations:** 1 Department of Biomechatronic Engineering, College of Biotechnology and Bioengineering, Sungkyunkwan University (SKKU), Suwon, South Korea; 2 Department of Surgery, Hangang Sacred Heart Hospital, College of Medicine, Hallym University, Seoul, South Korea

## Abstract

We report a cell-dispensing technique, using a core–shell nozzle and an absorbent dispensing stage to form cell-embedded struts. In the shell of the nozzle, a cross-linking agent flowed continuously onto the surface of the dispensed bioink in the core nozzle, so that the bioink struts were rapidly gelled, and any remnant cross-linking solution during the process was rapidly absorbed into the working stage, resulting in high cell-viability in the bioink strut and stable formation of a three-dimensional mesh structure. The cell-printing conditions were optimized by manipulating the process conditions to obtain high mechanical stability and high cell viability. The cell density was 1 × 10^7^ mL^−1^, which was achieved using a 3-wt% solution of alginate in phosphate-buffered saline, a mass fraction of 1.2 wt% of CaCl_2_ flowing in the shell nozzle with a fixed flow rate of 0.08 mL min^−1^, and a translation velocity of the printing nozzle of 10 mm s^−1^. To demonstrate the applicability of the technique, preosteoblasts and human adipose stem cells (hASCs) were used to obtain cell-laden structures with multi-layer porous mesh structures. The fabricated cell-laden mesh structures exhibited reasonable initial cell viabilities for preosteoblasts (93%) and hASCs (92%), and hepatogenic differentiation of hASC was successfully achieved.

Tissue engineering has been widely applied to the regeneration of damaged tissues and organs using a combination of cells, an engineered extracellular matrix (or scaffold), and appropriate bioactive growth and differentiation factors^1^,^2^,^3^. The scaffold has been shown to be an important factor in cell attachment, growth, and differentiation; however, the mechanisms for the effects of the chemical and biological compositions and the physical structures that are required to encourage proper tissue regeneration are not completely understood^4^.

Biomedical scaffolds for tissue engineering should possess various chemical and physical properties, including biocompatibility, with minimal cytotoxic effects to allow high cell attachment and proliferation; should be biodegradable; should have a highly porous structure (appropriate pore size, tortuosity, pore-interconnectivity) to enable easy vascularization and efficient transportation of nutrients and metabolic waste; and should have appropriate mechanical properties to endure the compressive and shear stresses from the micro-environmental conditions^5^,^6^,^7^.

Recently, scaffold-based tissue regenerative processes have focused on cell-printing strategies to fabricate cell-embedded scaffolds. Such printed scaffold can be more efficient and versatile in the homogeneous distribution of culturing cells in 3D structures than conventional scaffolds, and various cell-types can be printed in the desired region within the scaffold^8^,^9^,^10^. For this reason, cell-printing processes have been widely investigated. A variety of methods have been reported for directly embedding cells into matrix materials, including cell-plotting using pneumatic pressure, cell-printing using an inkjet printer, laser-guided direct/indirect printing, and predesigned molds^11^ to obtain optimal cell-embedded porous structures^12^,^13^,^14^. However, despite much work towards obtaining highly porous cell-laden structures, problems with mechanical properties remain, which lead to breakdown of the micro-internal porous structure, as well as long fabrication times and limited thickness of the cell-embedded scaffolds.

Here we describe a novel cell-printing process to obtain highly porous structures, with a thick cell-laden structure that has sufficient initial cell-viability, using a technique with a short processing time. We designed a new cell-printing method using a core–shell nozzle. A mixture of cells and alginate, which has been widely used as a cell-delivering agent due to the rapid gelation in the presence of calcium ions and good biocompatibility, flows in the core region; simultaneously, calcium chloride solution, which is used as a cross-linking agent of the alginate, flows in the shell region. Following contact between the alginate and Ca^2+^, the mixture of cells and alginate rapidly solidified.

The calcium chloride solution in the shell region results in cell damage due to the long contact time with the printed cells on the dispensing stage. For this reason, we used an absorbent stage to remove remnant calcium chloride solution during dispensing. We optimized the process parameters to provide cell-dispensing conditions to enable the formation of 3D porous structures that yielded good cell-viability. Using this technique, we were able to produce cell-laden structures with various dimensions, without requiring supplementary molds or any supporting synthetic polymers. To demonstrate the feasibility of the technique, we used preosteoblasts (MC3T3-E1) and human adipose stem cells (hASCs) to obtain 3D cell-laden mesh structures. The initial cell viability and proliferation of the cell-embedded structures were characterized, and hepatogenic differentiation of the hASC-laden mesh structure under hepatogenic medium was observed.

## Results and Discussion

### Stability of a cell-laden single line of three cell-dispensing methods

**Figure 1:**
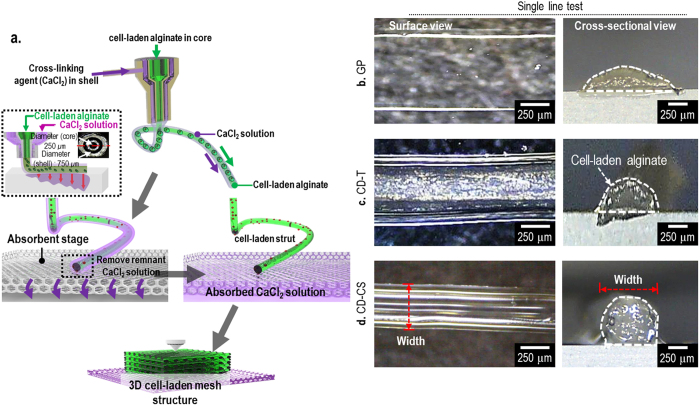
Schematic diagram showing the CD-CS method using a core–shell nozzle and single lines of cell-laden alginate. (**a**) The CD-CS cell-dispensing process with a nozzle with a 250-μm core and a 750-μm shell, where the cross-linking agent flowed in the shell and the cell-laden alginate flowed in the core. (**b**) A general process (GP) without cross-linking during dispensing. (**c**) The cell-dispensing process (CD-T) with an aerosol cross-linking process. (**d**) The CD-CS process. The schematic diagram was drawn by S.H. Ahn.

We compared the stability of the of the cell-laden struts fabricated using three processes: a general process (GP) without cross-linking during the dispensing process^15^, a cell-dispensing process (CD-T) assisted via aerosol-based cross-linking^16^,^17^, and a new cell-dispensing process (CD-CS) using a nozzle with a 250-μm core and a 750-μm shell, in which cross-linking agent flowed in the shell and a cell-laden hydrogel flowed in the core, as shown in Fig. 1(a). The pneumatic pressure of the mixture of 1 × 10^7 ^mL^−1^ of MC3T3-E1 cells in 3-wt% alginate was 70 kPa. With the CD-T process, the aerosol flow rate of the 5-wt% solution of CaCl_2_ was 1.4 mL min^−1^ and in the CD-CS process the flow rate of 1.2-wt% CaCl_2_ solution in the shell region was 0.08 mL min^−1^ 18. To investigate the stability of the cylindrical shapes, surface and cross-sectional views of single-line cell-laden struts were obtained using an optical microscope. Figure 1(b–d) show optical microscope images of the single struts fabricated using the GP, CD-T and CD-CS processes. The single strut formed using the CD-CS process exhibited a completely round cross-section (in contrast to those formed using the GP and CD-T processes), which is attributed to the rapid gelation of the alginate immediately following making contact with the cross-linking agent. Based on this result, we may conclude the CD-CS process is suitable for the formation of cylindrical struts.

### Cell-laden mesh structures fabricated using CD-CS with and without an absorbent dispensing stage

**Figure 2:**
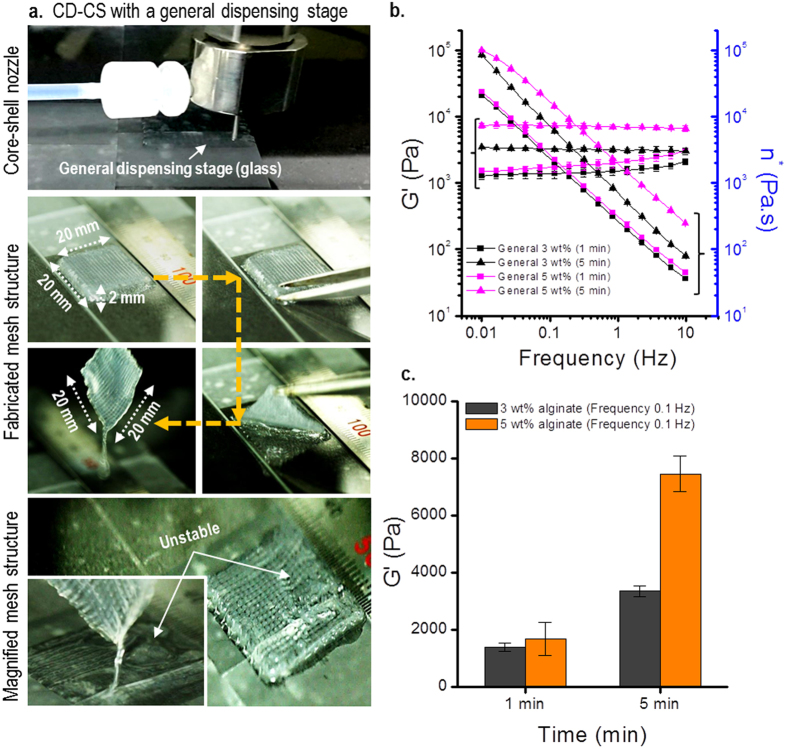
Unstable cell-laden structure and rheological properties. (**a**) Cell-laden mesh structure fabricated using the CD-CS process with a glass dispensing stage. (**b**) The storage modulus *G*′ and the complex viscosity n* of cell-laden alginate cross-linked on a glass dispensing stage with two different cross-linking times. (**c**) A comparison of the storage moduli at 0.1 Hz.

Because of the ability to fabricate cylindrical struts using the CD-CS process, we attempted to form cell-laden mesh structures. Biomedical scaffolds for tissue regeneration should be mechanically stable and exhibit highly porous 3D structures to enable cell-to-cell and cell-to-matrix interactions, encourage the formation of blood vessels, and to convey nutrients and remove metabolic waste^19^. Figure 2(a) shows the CD-CS cell-dispensing process using a glass dispensing stage. The optical images show the fabricated cell-laden mesh structure, which had dimensions of 20 × 20 × 2 mm^3^. This structure appeared to be a stable mesh structure; however, as shown in the magnified image, the cell-laden micro-struts were detached from each other, which may lead to an unstable porous mesh structure. Such instability of the micron-scale struts in the mesh structure was attributed to some cross-linking agent (i.e., CaCl_2_ solution) remaining in the dispensing stage during the fabrication process, which inhibits the formation of dispensed cylindrical cell-laden struts, as the inter-strut adhesive forces are weak. We observed the time-dependent modulus of the dispensed struts with the cross-linking agent using a glass dispensing stage to test the rheological properties. Figure 2(b) shows the complex viscosity n* and storage modulus *G*′ of the 3- and 5-wt% cell-laden alginates with two cross-linking times, i.e., 1 min and 5 min, and with a CaCl_2_ solution concentration of 1.2 wt%. The storage modulus was significantly larger with the longer cross-linking time, as shown in Fig. 2(c). These rheological results show that the cell-laden struts became harder as the processing time increased, reducing the inter-strut adhesion, and possibly leading to a decrease in the activities of the embedded cells.

**Figure 3:**
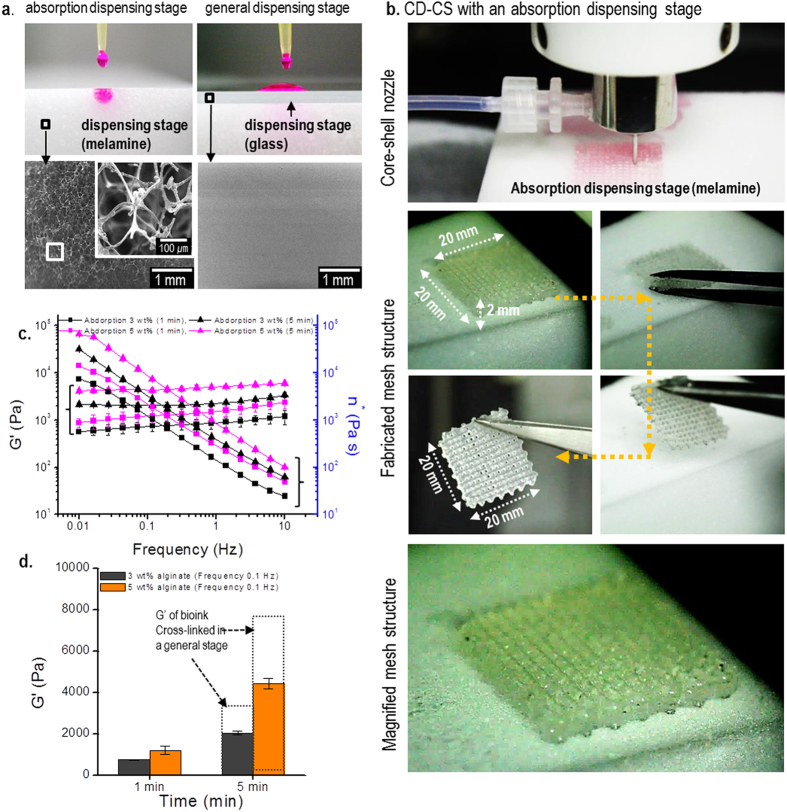
The CD-CS process with an absorbent dispensing stage and the resulting stable cell-laden mesh structure. (**a**) Optical and SEM images of the absorbent dispensing stage and the glass dispensing stage. The wetted red dye shows the water absorption properties of the stages. (**b**) A cell-laden mesh structure fabricated using the CD-CS process with an absorbent stage. (**c**) The storage modulus *G*′ and complex viscosity n* of the cell-laden alginate cross-linked on the absorbent dispensing stage. (**d**) A comparison of the storage moduli at 0.1 Hz.

To overcome this problem, we designed a modified CD-CS system with a new dispensing stage, which was highly water-absorbing. This absorbent dispensing stage was formed of the thermosetting polymer melamine resin, and the surface was highly porous, as shown in Fig. 3(a). On this absorbent stage, we dispensed the cell-laden alginate solution in the core region and the cross-linking agent in the shell region. Figure 3(b) shows the cell-dispensing process with the absorbent dispensing stage and optical images showing the fabricated mesh structure. As shown in the optical images, a highly porous cell-laden structure was well obtained due to the rapid absorption of the cross-linking agent onto the dispensing stage. To evaluate the effects of the absorbent stage on the modulus of the dispensed struts, we measured the modulus of the cell-laden solution with two cross-linking times (i.e., 1 min and 5 min). Figure 3(c) shows the rheological properties with 3- and 5-wt% cell-laden alginate suspensions for the two cross-linking times, and with a 1.2-wt% CaCl_2_ solution. As with the previous results, the storage modulus of the cell-laden alginate with the longer cross-linking time was larger; however, the difference in the moduli between the two cross-linking times was significantly smaller than that obtained using the glass dispensing stage, as shown in Fig. 3(d). This result shows that the cross-linking agent was rapidly absorbed into the dispensing stage, resulting in increased adhesion between the struts due to the viscosity of the partially cross-linked alginate.

### Detection of stable processing conditions of CD-CS

Although it is known that a low mass-fraction of the cross-linking agent (i.e., CaCl_2_) is not especially toxic to the cells embedded in alginate-based bioink, only a small quantity of the agent should be used in any cell-dispensing process. To achieve a quantity of CaCl_2_ solution that satisfies both the porous structural formation and provides high viability of the cells embedded in the bioink, we measured the stability of the cylindrical shapes formed by the cell-laden alginate struts as a function of the mass fraction of CaCl_2_ solution, with a constant flow rate of the solution in the shell region. To observe the shape of the dispensed alginate struts on the absorption stage, we investigated the ratio of the long axis *a* to the short axis *b* using optical microscopy. During these tests, the alginate concentration was varied in the range 3–5 wt% in PBS, the processing temperature was fixed at 28 ± 3 °C, and the flow rate of CaCl_2_ solution was fixed at 0.08 mL min^−1^.

**Figure 4:**
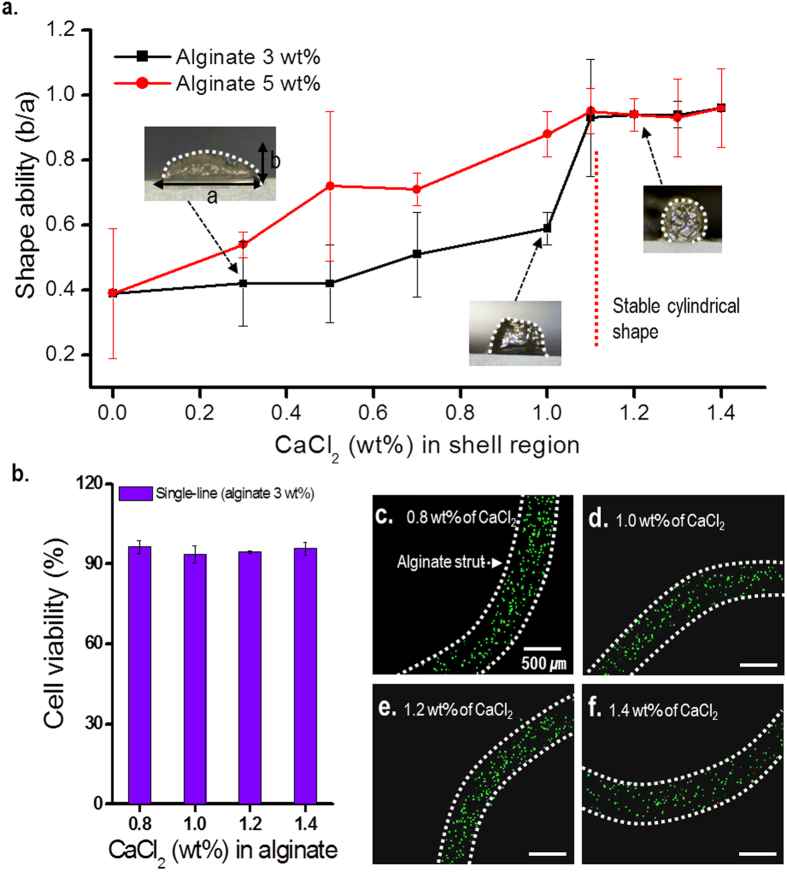
Optimum conditions for the CD-CS process. (**a**) The stability of the cylindrical cell-laden alginates (3 and 5 wt%) for various calcium chloride solutions. (**b**) The cell-viability of the cell-laden struts formed with a 3-wt% concentration of alginate. Fluorescence images showing the cell viability for various CaCl_2_ solutions in shell nozzle, (**c**) 0.8, (d) 1.0, (**e**) 1.2, and (**f**) 1.4 wt%. In these images, the live cells are shown in green and dead cells shown in red.

Figure 4(a) shows the effects of various mass factions of CaCl_2_ in the shell nozzle on the shape of the cell-laden alginate strut. A concentration of CaCl_2_ greater than 1.1 wt% in the shell region resulted in a stable cylindrical shape of the extruded struts, with *b*/*a *> 0.8. From this result, we may conclude that concentrations of CaCl_2_ greater than 1.1 wt% in the absorbent dispensing stage result in the formation of stable cylindrical cell-laden alginate struts, regardless of the limited mass fraction of the alginate-based bioink. The optical images shown in Fig. 4(a) reveal a stable cylindrical form of the dispensed bioink struts for solutions with a CaCl_2_ concentration greater than 1.2 wt%, whereas the cross-sectional image of the alginate strut, which was fabricated with a lower concentration of CaCl_2_ solution, did not maintain a stable cylindrical shape.

The initial cell-viability following dispensing of cells is a significant factor for cell-embedded materials, because the cell viability can affect cellular responses (i.e., migration, growth, and differentiation) after long culture periods^15^. To measure the cell viability in the cell-laden struts after dispensing, the dispensed cell-alginate struts were stained using calcein-AM (to identify live cells) and ethidium homodimer-1 (to identify dead cells). To investigate the effect of the mass fraction of CaCl_2_ in the shell region on the cell-viability, we prepared samples with mass fractions of CaCl_2_ of 0.8, 1.0, 1.2 and 1.4 wt% with the same processing conditions (i.e., a flow rate of CaCl_2_ solution of 0.08 mL min^−1^, a translation speed of the nozzle of 10 mm s^−1^, and a pneumatic pressure of 70 kPa in the core region). In these experiments, the embedded cells had a density of 1.0 × 10^7^ cells mL^−1^, and the concentration of alginate was fixed at 3.0 wt%. Figure 4(b) shows the cell-viability of the cell-laden alginate after 1 day on the absorbent dispensing stage. For all mass fractions of the cross-linking agent, the cell viability was greater than 92%, although the mass fraction of the agent in the shell region increased. Figure 4(c–f) show fluorescence images showing the cell-viability. The CD-CS system with an absorbent dispensing stage cross-linking process with 1.4 wt% of CaCl_2_ flowing in the shell nozzle did not adversely affect the viability to the embedded cells.

### 3D mesh structure of MC3T3-E1-laden bioink and cellular activities

**Figure 5:**
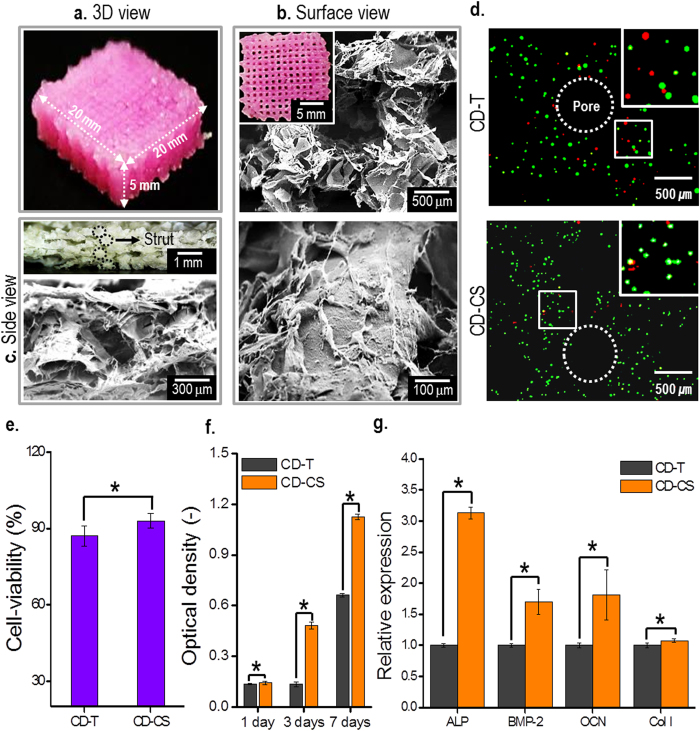
Optical and SEM images and cellular activities of the 3D cell-laden mesh structures. Optical and SEM images of 3D MC3T3-E1-laden mesh structures fabricated using CD-CS. (**a**) 3D view, (**b**) surface view, and (**c**) side view. (**d**) Fluorescence images showing the cell viability of the cell-laden mesh structures fabricated using the CD-T and CD-CS after 1 day. (**e**) Cell viability measured using the fluorescence images and (**f**) MTT assay results showing cell proliferation. (**g**) Relative expression of ALP, BMP-2, OCN, and Col-I gene expression levels in MC3T3-E1 cells for 14 days. In the fluorescence images, the live cells are shown in green and dead cells shown in red. Asterisks (*) indicate *p *< 0.05.

To obtain the 3D MC3T3-E1-laden mesh structures, stable processing conditions (i.e., a cell density of 1 × 10^7^ mL^−1^, a 3-wt% alginate solution in PBS, a pneumatic pressure of 70 kPa in the core region of the nozzle, a CaCl_2_ flow rate of 0.08 mL min^−1^ in shell region of the nozzle, and translation speed of 10 mm s^−1^) were used with CD-CS with the absorbent stage. Figure 5(a–c) show optical microscope and scanning electron microscope (SEM) images of the fabricated cell-laden multi-layered mesh-structure (with dimensions of 20 × 20 × 5 mm^3^) in which the strut diameter was 414 ± 39 μm and pore size between the struts was 527 ± 28 μm.

To observe the cell viability following fabrication of cell-laden mesh structures, the cells were analyzed using fluorescence microscopy, as shown in Fig. 5(d). As a control, we used the cell-laden structure fabricated using the CD-T process^18^. The live cells after 1 day were homogeneously distributed in the struts, and the cell viabilities for the cell-laden structures fabricated using the CD-T process was 87 ± 4% and the viability of the structures fabricated using the CD-CS process was 93 ± 3%, as shown in Fig. 5(e). Using the MTT assay, we measured the proliferation of viable cells, as shown in Fig. 5(f). The proliferation of viable cells was significantly greater in the cell-laden mesh structure fabricated using the CD-CS process than with that fabricated using the CD-T process. The differences in the cell-viability and proliferation between the processes were significant (p < 0.05), and we attribute this to differences in the contact times of the CaCl_2_ solution and cells in the cell-laden structure. With the CD-CS process with the absorbent stage, the cross-linking agent was immediately absorbed into the stage, whereas with the CD-T process, aerosols from the cross-linking agent were attached to the struts throughout the fabrication process of the cell-laden mesh structure. It follows that the CD-CS process is safer than the CD-T process. To evaluate osteogenic differentiation of the cell-laden structures for cell culture period (14 days), osteogenesis-related genes (alkaline phosphatase (ALP) activity, bone morphogenetic protein-2 (BMP-2), osteocalcin (OCN), and collagen-I (Col-I)) were measured by RT-PCR. Figure 5(g) demonstrates the expression of genes involved in osteoblastic differentiation of MC3T3-E1 cells laden in the structures fabricated by CD-T and CD-CS process. The levels of ALP, BMP-2, OCN, and Col-I expression in the structure fabricated by CD-CS process were meaningfully higher than those in the structure fabricated by CD-T process. The expression of specific genes related to osteoblastic differentiation can suggest that the cell-laden structure fabricated using the CD-CS process provided biological conditions more favorable for MC3T3-E1 than did the structure fabricated using CD-T process.

### Application of the CD-CS process to fabricate the hASC-laden structures and hepatogenic differentiation of hASCs in the structure

Human adipose-derived stem cells (hASCs) have been widely used in cell therapy and tissue regeneration applications due to the multi-potent and effective inducement into a variety of cell types^20^. Bio-artificial liver hepatogenic differentiation of hASCs has attracted much recent research attention. Wang *et al.* reported hASCs loaded onto a porous poly-lactide-co-glycolide scaffold to examine cell hepatogenesis^21^. The cells were well proliferated in a hepatic-inducing medium, and differentiated into functional hepatocytic phenotypes. In this section, to demonstrate the feasibility of the CD-CS fabrication technique, we fabricate 3D porous mesh structures with embedded hASCs and used the hASCs-laden structure fabricated by CD-T as a control.

**Figure 6:**
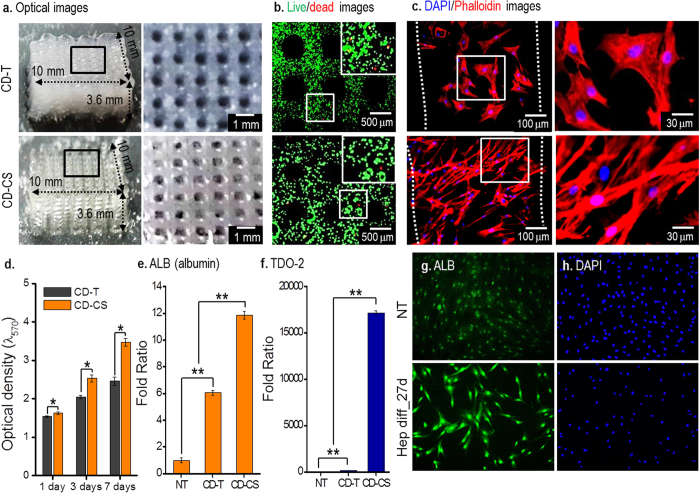
Optical and fluorescence images of the hASC-laden mesh structures fabricated using the CD-T and CD-CS method and hepatogenic differentiation. (**a**) Optical images of the mesh structures. (**b**) Fluorescence images showing live cells in green and dead cells in red after 1 day. (**c**) DAPI/phalloidin staining after 27 days of culture of the mesh structure. (**d**) MTT result of the cell-laden structures. Expression levels of hepatocyte-specific genes (**e**) albumin and (f) TDO-2 using real-time RT-PCR on day 27 (*n *= 3, mean* *± SD, statistical comparison was performed using Student’s t-test, *p *< 0.01(**)) and (**g,h**) immunofluorescence images.

The fabrication conditions and mass fraction of alginate were the same as those used to fabricate the preosteoblast-laden mesh structure shown in Fig. 5. The cell density of hASCs in the mesh structure was 1 × 10^7^ mL^−1^. Figure 6(a) shows an optical microscope image of the cell-laden structures, revealing a highly porous structure, with dimensions of 10 × 10 × 3.6 mm^3^. Figure 6(b) shows fluorescence images, showing that the cell viability after 1 day in the porous structure fabricated using CD-CS was 92.3 ± 3%, while the structure using CD-T was 89.6%. Figure 6(c) shows DAPI/Phallodin images after 27 days and those demonstrate the hASCs were more widely distributed on the structure fabricated by CD-CS than that of CD-T process. Figure 6(d) shows the proliferation of viable hASCs, which demonstrate that the cells in the structure fabricated by CD-CS were well alive and exhibited metabolic function during the culture period compared with the structure fabricated by CD-T.

To examine the differentiation of the cell-laden structures, albumin (ALB) and tryptophan 2,3-dioxygenase (TDO2) were measured after 27 days of culturing in the hepatogenic medium, as shown in Fig. 6(e,f). In the result, ‘NT’ means undifferentiated hASCs, which were not treated with hepatogenic medium. The hASCs from the fabricated structures were found to express liver-specific genes. However, the level of liver-specific genes in the structure fabricated by CD-CS demonstrated much significantly higher than those in the structure fabricated by CD-T process. This result means that the cell-laden structure fabricated using the CD-CS process provided more favorable hepatogenic differentiation condition for hASCs than did the structure fabricated using CD-T process. Figures 6(g,h) show the fluorescence images of differentiated hASCs in the structure fabricated by CD-CS and undifferentiated hASCs (NT).

These results show that the fabrication process (CD-CS) provides a medium with favorable cell viabilities for preosteoblasts and hASCs, and the cell-laden structure is appropriate for hepatogenesis of hASCs.

## Conclusion

We have described a novel cell-dispensing method using a core–shell nozzle, whereby the core dispenses a cell–alginate mixture and the shell dispenses a cross-linking agent, together with an absorbent dispensing stage. By controlling the mass fraction of calcium chloride solution in the shell of the nozzle, we successfully obtained highly porous cell-laden mesh structures with a cell-viability of approximately 93% for preosteoblasts (MC3T-E1). The embedded cells survived well and proliferated during the cell culture period. Furthermore, the cells in the 3D structure exhibited proper metabolic function during the culture period. In addition, an hASC-laden mesh structure was fabricated with the same process conditions, and exhibited a cell viability of 92.3 ± 3%. Following 27 days of culturing a hepatogenic medium, ALB and TDO2 were measured, and we found that liver-specific genes in the structure were significantly expressed. The ability to fabricate highly porous structures with controllable thickness shows that this bio-fabrication method has significant potential for tissue engineering and the development of cell-laden structures.

## Experimental

### Materials and cell-mixture

Preosteoblast cells (MC3T3-E1; ATCC, Manassas, VA, USA) and human adipose-derived stem cells (hASCs; Anterogen corporation, South Korea) were used in the cell-printing process. Low-viscosity, high-G-content LF10/60 alginate (FMC BioPolymer, Drammen, Norway) was used as the cell-supporting material. The mixture of cells and alginate (3 wt% in PBS) was mixed using a three-way stopcock tool at a density of 1 × 10^7^ cells mL^−1^.

### Rheological measurements of the cell-mixtures

The rheological properties (i.e., the complex viscosity n*, storage modulus *G*′ and loss modulus *G*″) of the cell-laden alginate solution were evaluated using a rotational rheometer (Bohlin Gemini HR Nano, Malvern Instruments, Surrey, UK) equipped with cone-and-plate geometry (40 mm in diameter; with a cone angle of 4° and a gap of 150 μm). A dynamic frequency sweep in the range 0.01–10 Hz was carried out with 1% strain and at 27 °C within the linear viscoelastic region.

### Fabrication of a cell-laden mesh structure

A computer-controlled three-axis robot system (DTR2-2210T, Dongbu Robot, Bucheon, South Korea) was used with the dispenser to obtain layer-by-layer printed bioink structures. A humidifier (Tess-7400; Paju, South Korea) was used during the aerosol cross-linking process. The pneumatic pressure in the dispensing process was 70 ± 10 kPa. Each layer of the mixture of cells and alginate was dispensed on top of the existing one, in the planar direction perpendicular to the lower layer, forming a mesh structure.

### Characterisation of the 3D structure fabricated using the cell-laden alginate

The surface morphology of the 3D structures was visualised using an optical microscope (Model BX FM-32; Olympus, Tokyo, Japan) and a SEM (SNE-3000M, SEC, Inc., South Korea). The pore size of the structures was defined as the separation between the parallel struts.

The compressive mechanical properties were characterized using a universal testing machine (Top-tech 2000; Chemilab, Seoul, South Korea) in compressive mode. Stress–strain curves were recorded at a compression rate of 0.05 mm s^−1^ with a sample that was 6 mm in diameter and 1.3-mm-thick. All data were expressed as the mean plus/minus the standard deviation, and the measurements were repeated five times.

### *In-vitro* cell culture and MTT assay

The structures were dispensed using the MC3T3-E1 and ASCs and were cultured and maintained in α-containing 10% FBS and 1% antibiotic (antimycotic; Cellgro, Mediatech, Manassas, VA). The structures were incubated in an atmosphere of 5% CO_2_ at 37 °C, and the medium was changed every second day. The proliferation of viable cells was determined using the MTT cell proliferation assay (Cell Proliferation Kit I; Boehringer Mannheim). The assay was based on the cleavage of the yellow tetrazolium salt, MTT, via mitochondrial dehydrogenases in viable cells to produce purple formazan crystals. Cell-laden structures were incubated in a 0.5-mg mL^−1^ MTT solution for 4 h at 37 ^o^C. The absorbance at 570 nm was measured using a microplate reader (EL800; Bio-Tek Instruments). Four samples were tested for each incubation period, and each of the tests was performed in triplicate.

### Real-Time RT-PCR Analysis

The expression of osteogenesis-related genes (ALP, BMP-2, OCN, and Col-I) was examined using real-time quantitative RT-PCR (qRT-PCR). Total RNA was first extracted using the TRIzol reagent (Invitrogen) according to the manufacturer’s protocol. First-strand cDNA synthesis was then performed using reaction mixtures containing 1-μL (200 units) SuperScript II reverse transcriptase (Invitrogen), 1-μg total RNA, 50-ng oligo (dT)_20_ primer, 1 × reaction buffer, and a 0.5 × 10^−3^ m deoxynucleotide triphosphate mixture for 1 h at 42 °C and was stopped by incubation at 70 °C for 15 min. Finally, the cDNA was diluted 1:10 with distilled water and used for real-time RT-PCR with the iQ SYBR Green Supermix (Bio-Rad) in an iCycler iQ (Bio-Rad). GAPDH was used as an internal control to normalize transcript levels. Each experiment was repeated three times. Gene-specific primers are listed in the reference^22^.

### Live/dead cell assay

To measure the cell-viability of the dispensed single line struts and the 3D structures, the structure was exposed to 0.15-mM calcein-AM, and-2 mM ethidium homodimer-1 for 45 min in an incubator. The stained structure was then analysed using a microscope (TE2000-S; Nikon, Tokyo, Japan) equipped with an epifluorescence attachment and a SPOT RT digital camera (SPOT Imaging Solutions, Sterling Heights, MI). To evaluate the initial cell viability after 1 day, the numbers of live and dead cells were counted using the ImageJ software package (NIH, Bethesda, MD). The ratio of the number of live cells to the number of total cells (i.e., live and dead cells) was calculated, and this ratio was normalised to the cell viability determined using trypan blue (Mediatech, Herndon, VA) before dispensing the cell-laden alginate.

### *In-vitro* differentiation of ASCs to hepatocytes

Human ASCs were cultured in low-glucose DMEM (cat# SH30021.01, Hyclone) containing 0.1% gentamicin and 10% FBS. The hASC-laden alginate structures were incubated in basal medium (low-glucose DMEM containing 0.1% gentamicin and 10% FBS). After 1 day, the 3D structure were washed using HBSS twice and replaced with low-glucose DMEM containing 0.1% gentamicin, 2% FBS, 20-ng/ml EGF (CYT-217, ProSpec), 10-ng/ml bFGF (CYT-218, ProSpec), and cultured for a further 2 days. The media was then replaced with low-glucose DMEM containing 0.1% gentamicin, 2% FBS, 1× ITS (Insulin-Transferrin-Selenium Supplement, 100×), 10-ng/ml OSM (CYT-231, ProSpec), 10-ng/ml bFGF, 20-ng/ml HGF(CYT-244, ProSpec), 1-μM dexamethasone, 5-mM nicotinamide, and 0.1% DMSO. The medium was replaced every 2–3 days. Hepatogenic differentiation was assessed using RT-PCR analysis of liver-specific genes.

### RNA isolation and quantitative real-time PCR analysis to measure hapatogenic genes

The total RNA from the cultured cell-laden structure was isolated using Trizol reagent (Life technologies, Inc., Carlsbad, CA) according to the manufacturer’s instructions. The gene-specific primers were as follows: human albumin (ALB) (forward: 5′-GTCACCAAATGCTGCACAGA-3′, reward: 5′-ACGAGCTCAACAAGTGCAGT-3′), human tryptophan 2,3- dioxygenase (TDO2) (forward: 5′-GTGTGCATGGTGCACAGAAT-3′, reward: 5′-GGGTTCATCTTCGGTATCCA-3′) and housekeeping gene human β-actin (forward: 5′-GTCCTCTCCCAAGTCCACAC-3′, reward: 5′-GGGAGACCAAAAGCCTTCAT-3′). All amplifications were carried out in 20 μl of pre-mixture containing 500 nmol/l of gene-specific primers, 2× SYBR and 6 μl of template, with the following conditions: denaturation at 95 °C for 5 min, followed by 40 cycles of 95 °C for 10 sec, 58 °C for 15 sec, and 72 °C for 15 sec, with a final extension at 72 °C for 5 min. The reactions were carried out in Roche LC480.

### hASCs staining for immunofluorescence microscopy

To carry out immunofluorescence staining, the cells from the cell-laden structure were placed on a cover slip. The cells were washed twice with PBS, fixed in 4% PFA, washed with PBS, and then incubated for 3 min in 0.1% (v/v) Triton X-100 in PBS. The cells were then incubated using blocking solution (1:10, ab126587, abcam) for 30 min and 0.05% TBS-T, containing antibodies against albumin (1:100, ab135575, abcam), for 1.5 hrs at room temperature. After washing with 0.05% TBS-T in PBS, cy2-conjugated fluorescent secondary antibodies (Jackson ImmunoResearch) and DAPI were added at dilutions of 1:200. The cells were then washed with 0.05% TBS-T three times and imaged using a fluorescence microscope (Olympus IX81, Japan).

### Statistical analysis

Data presented are expressed as means ± SD. Single-factor analysis of variance (ANOVA) was used as the statistical test, and the significance level was set at *p *< 0.05 (*).

## Additional Information

**How to cite this article**: Ahn, S.H. *et al.* A novel cell-printing method and its application to hepatogenic differentiation of human adipose stem cell-embedded mesh structures. *Sci. Rep.* **5**, 13427; doi: 10.1038/srep13427 (2015).
